# Insecticide resistance status of *Aedes aegypti* and *Aedes albopictus* mosquitoes in Papua New Guinea

**DOI:** 10.1186/s13071-019-3585-6

**Published:** 2019-07-03

**Authors:** Samuel Demok, Nancy Endersby-Harshman, Rebecca Vinit, Lincoln Timinao, Leanne J. Robinson, Melinda Susapu, Leo Makita, Moses Laman, Ary Hoffmann, Stephan Karl

**Affiliations:** 1 Vector-Borne Diseases Unit, PNG Institute of Medical Research, P.O. Box 378, Madang, 511 Madang Province Papua New Guinea; 2 School of BioSciences, Bio21 Institute, The University of Melbourne, 30 Flemington Rd., Parkville, VIC 3010 Australia; 3 Australian Institute of Tropical Health and Medicine, James Cook University, 1/14-88 McGregor Road, Smithfield, QLD 4870 Australia; 4 Burnet Institute, 85 Commercial Road, Melbourne, VIC 3004 Australia; 5 National Department of Health, Waigani Drive, P.O. Box 807, Port Moresby, Papua New Guinea

**Keywords:** *Aedes aegypti*, *Aedes albopictus*, Pyrethroid resistance, Deltamethrin, Papua New Guinea, Port Moresby, Madang, Bioassay, Insecticide

## Abstract

**Background:**

*Aedes aegypti* and *Ae. albopictus* are important vectors of infectious diseases, especially those caused by arboviruses such as dengue, chikungunya and Zika. *Aedes aegypti* is very well adapted to urban environments, whereas *Ae. albopictus* inhabits more rural settings. Pyrethroid resistance is widespread in these vectors, but limited data exist from the Southwest Pacific Region, especially from Melanesia. While *Aedes* vector ecology is well documented in Australia, where incursion of *Ae. albopictus* and pyrethroid resistance have so far been prevented, almost nothing is known about *Aedes* populations in neighbouring Papua New Guinea (PNG). With pyrethroid resistance documented in parts of Indonesia but not in Australia, it is important to determine the distribution of susceptible and resistant *Aedes* populations in this region.

**Methods:**

The present study was aimed at assessing *Aedes* populations for insecticide resistance in Madang and Port Moresby, located on the north and south coasts of PNG, respectively. Mosquitoes were collected using ovitraps and reared in an insectary. Standard WHO bioassays using insecticide-treated filter papers were conducted on a total of 253 *Ae. aegypti* and 768 *Ae. albopictus* adult mosquitoes. Subsets of samples from both species (55 *Ae. aegypti* and 48 *Ae. albopictus*) were screened for knockdown resistance mutations in the voltage-sensitive sodium channel (*Vssc*) gene, the target site of pyrethroid insecticides.

**Results:**

High levels of resistance against pyrethroids were identified in *Ae*. *aegypti* from Madang and Port Moresby. *Aedes albopictus* exhibited susceptibility to pyrethroids, but moderate levels of resistance to DDT. Mutations associated with pyrethroid resistance were detected in all *Ae*. *aegypti* samples screened. Some genotypes found in the present study had been observed previously in Indonesia. No *Vssc* mutations associated with pyrethroid resistance were found in the *Ae. albopictus* samples.

**Conclusions:**

To our knowledge, this is the first report of pyrethroid resistance in *Ae*. *aegypti* mosquitoes in PNG. Interestingly, usage of insecticides in PNG is low, apart from long-lasting insecticidal nets distributed for malaria control. Further investigations on how these resistant *Ae*. *aegypti* mosquito populations arose in PNG and how they are being sustained are warranted.

**Electronic supplementary material:**

The online version of this article (10.1186/s13071-019-3585-6) contains supplementary material, which is available to authorized users.

## Background

*Aedes aegypti* and *Ae. albopictus* are vectors of important neglected infectious diseases, especially those caused by arboviruses such as dengue, chikungunya and Zika. They are known to be invasive and have expanded to several new regions relatively recently [1–5].

Insecticide resistance, especially against pyrethroids, is a major threat to vector-borne disease control worldwide. *Aedes aegypti* is well-adapted to urban habitats, and as a result, is usually more likely to be exposed to insecticides and develop resistance than is *Ae*. *albopictus* [6].

In the Asia-Pacific Region, reports have confirmed pyrethroid resistance in *Aedes* populations in many countries [7], and there is a geographical divide between regions where *Aedes* spp. are still susceptible (mainly Australia [8]) and those where *Aedes* spp. are resistant [8]. *Aedes albopictus* has not established populations in Australia so far [9].

While there are studies from parts of Indonesia indicating pyrethroid resistance in *Ae*. *aegypti* [10], Papua New Guinea (PNG) is one of the missing links in the region, where vector distribution and insecticide resistance status are almost completely unknown. While information about the vectors is very scarce [5], it is known that arboviruses represent a significant health burden in PNG; dengue viruses 1–4 circulate in the country and chikungunya outbreaks have been documented [11–13]. Due to the size of PNG, as well as its central location in the South Pacific Region, rapid population growth and increasing economic importance, understanding *Aedes* vector biology and resistance status in PNG is likely to be relevant to vector-control efforts in the South Pacific Region more generally. For instance, *Aedes* mosquitoes (and their resistance genes) may be transported from PNG to neighbouring countries, including Australia. Mosquito incursions to Australia are intercepted on a regular basis [14]. *Aedes aegypti* is present both in the Torres Strait and on the Australian mainland, but no phenotypic resistance or voltage-sensitive sodium channel (*Vssc*) resistance mutations have been detected in mosquitoes from these locations [8]. Although *Ae. albopictus* have not yet colonised the Australian mainland, they are present in the Torres Strait and PNG likely provides an important incursion route for this species [9, 15].

PNG exhibits a highly complex geography and a low level of urbanisation, with an estimated 13% of the population residing in urban areas [16]. However, this is rapidly changing as the expansion of urbanised areas, urban drift and anthropogenic environmental transformation are resulting in an increased proportion of humans at high risk from *Aedes* vector borne diseases. There is no widespread usage of insecticides in PNG in the public sector, apart from mass distribution of long-lasting insecticidal nets (LLIN) for malaria control since around 2009 [17], although large cities such as Port Moresby are not included in LLIN mass distributions. The LLIN distributed in PNG are treated with deltamethrin, which is a type 2 pyrethroid. In the private sector, insecticide usage is also limited, but not well documented. Large-scale commercial agriculture is not widespread and only a very small proportion of the available land (*c.*2.5%) is commercially farmed. Mining companies use fogging and indoor residual spraying (IRS) to protect their workforce from malaria. Hotels and businesses also engage in small-scale vector control activities, usually by hiring local pest control services. As such, there is potential for insecticide pressures in some areas in PNG to be underestimated.

The aim of this study was to generate initial information about *Aedes* vectors in PNG and their resistance levels to begin to address the knowledge gaps around these species. We collected *Aedes* mosquitoes from two population centres, Madang and Port Moresby, to determine species distribution and insecticide resistance status using bioassays to determine phenotype and DNA-based techniques to detect genetic markers of resistance.

## Methods

### Sampling sites and sample collection

Mosquitoes were collected as eggs and larvae in ovitraps placed in different locations around the provincial capital of Madang and the national capital of Port Moresby. In Madang, ovitraps (*n* = 15–20) were placed in the same locations every month for 12 months from February 2018 to January 2019. In Port Moresby traps were only placed once in December 2018 (*n* = 20). In both locations, traps were spread out across a large area in residential properties and hotel grounds, protected from rain. Trap contents were collected 5–7 days after trap placement.

### Mosquito rearing and bioassays

Mosquitoes were reared to adult stage in a permanent (Madang) or temporary (Port Moresby) insectary. Bioassays were conducted as previously described [18] using WHO standard methodology, and limiting concentration filter papers obtained from School of Biological Sciences, Universiti Sains Malaysia, which is the regional centre manufacturing these commodities. Assays were always accompanied by at least one control of 20 mosquitoes (20 per cylinder). Where possible each test included 20 mosquitoes per assay (20 per cylinder). Ideally, we conducted 4 assays and 2 controls per insecticide in parallel; however, this was not always possible given the limited mosquito numbers. The average humidity in the assays was 76.1% (range: 67.8–82.3%) and the average temperature was 27.03 °C (range: 22.6–32.0 °C). In Madang, PNG, day length varies from approximately 12.35 h in winter to 13.10 h in summer (southern hemisphere).

Insecticides used in this study were deltamethrin (0.05%), DDT (4%) lambdacyhalothrin (0.05%), bendiocarb (0.1%) and malathion (5%). *Aedes aegypti* and *Ae*. *albopictus* were exposed to the filter papers as per standard protocol for 60 min, and then removed, and placed into holding cups with access to sugar solution for 24 h. Outcome variables were the proportion of mosquitoes knocked down after 60 min and the proportion of mosquitoes dead after the 24 h holding period. Since DDT has been shown to act more slowly on anophelines, we also assessed mortality 48 h after exposure [19].

In PNG, *Ae. albopictus* and *Ae. aegypti* occur mixed in containers and separation of live larvae or adults prior to conducting the bioassays is not possible. Therefore, we conducted the assays with mixed species and identified the adults morphologically after the assays.

### Molecular analysis of kdr mutations

DNA was extracted from whole female mosquitoes using the High Pure PCR Template Preparation Kit (Roche, Millers Point, NSW, Australia) and suspended in 200 µl elution buffer. A 1:5 DNA dilution in PCR-grade H_2_O was prepared.

TaqMan® (Life Technologies Corporation, Mulgrave, VIC, Australia) assays were used to identify mutations in codons 989, 1016 and 1534 in the *Vssc* gene (numbered according to sequence of the most abundant splice variant of the house fly, *Musca domestica*) of *Ae*. *aegypti*. Primer and probe sequences used are as follows: Codon 989, forward primer (5′-TTC ATG ATC GTG TTC CGG GTA TT-3′), reverse primer (5′-ACG TCA CCC ACA AGC ATA CAA T-3′), probe (wildtype) (5′-CCC ACA TGG ATT CGA T-3′), probe (mutant) (5′-CCA CAT GGG TTC GAT-3′); codon 1016, forward primer (5′-CGT GCT AAC CGA CAA ATT GTT TCC-3′), reverse primer (5′-ATG AAC CGA AAT TGG ACA AAA GCA A-3′), probe (wildtype) (5′-AGA AAA GGT TAA GTA CCT GTG CG-3′), probe (mutant) (5′-AAG GTT AAG TCC CTG TGC G-3′); codon 1534, forward primer (5′-TCT ACA TGT ACC TCT ACT TTG TGT TCT TCA-3′), reverse primer (5′-GAT GAT GAC ACC GAT GAA CAG ATT C-3′), probe (wildtype) (5′-AAC GAC CCG AAG ATG A-3′), probe (mutant) (5′-ACG ACC CGC AGA TGA-3′). A 7 µl PCR reaction contained 40× TaqMan® assay (0.17 µl), 2× KAPA Fast PCR Probe Force qPCR Master Mix (KAPA Biosystems Inc.) (3.50 µl), ddH_2_O (1.33 µl) and genomic DNA (1:5 dilution) (2 µl).

Assays for each *Vssc* mutation were run in triplicate on a LightCycler® II 480 (Roche, Millers Point, NSW, Australia) instrument in 384-well plates with a pre-incubation of 3 min at 98 °C (ramp rate 4.8 °C/s) followed by 40 cycles of amplification at 95 °C for 10 s (2.5 °C/s ramp rate) and 60 °C for 20 s (2.5 °C/s ramp rate) (acquisition mode: single) with a final cooling step of 37 °C for 1 min (2.5 °C/s ramp rate). Endpoint genotyping was conducted for each mutation site (Roche LightCycler® 480 Software Version 1.5.1.62).

Mutations at *Vssc* codon 1534 in S6, Domain III of *Ae*. *albopictus* were screened using a forward primer of our own design (Alb171F: 5’-CCG ATT CGC GAG ACC AAC AT-3’) and the reverse primer of Kasai et al. (2011) (aegSCR8) [20]. Primer Alb171F was designed in the exon that contains *Vssc* codon 1534 in order to alleviate problems we encountered in sequencing across the intron. A 25 µl PCR mix included final concentrations of ThermoPol buffer Mg-free (1×) (New England Biolabs, Ipswich, MA, USA), dNTPs (200 µM each), MgCl_2_ (1.5 mM), 0.5 µM each of forward and reverse primers, 0.625 units of Immolase™ Taq polymerase (Bioline, London, UK), 2 µl genomic DNA (Chelex® extraction) diluted 1:10 and PCR-grade H_2_O to a final volume of 25 µl.

PCR conditions used to amplify the region were an initial denaturation at 95 °C for 10 min, 35 cycles of 95 °C for 30 s, annealing at 52 °C for 45 s and extension at 72 °C for 45 s, followed by a final extension step of 5 min at 72 °C and a hold at 10 °C.

PCR amplicons (220 bp) were sent to Macrogen Inc. in Seoul, Korea, for sequencing on a 3730xl DNA analyser. Sequences (up to 180 bp) were aligned and analysed using Geneious® 11.1.4 (Biomatters Ltd.) and mapped to a reference sequence (GenBank: KC152046.1).

## Results

### Overall species distribution

**Fig. 1:**
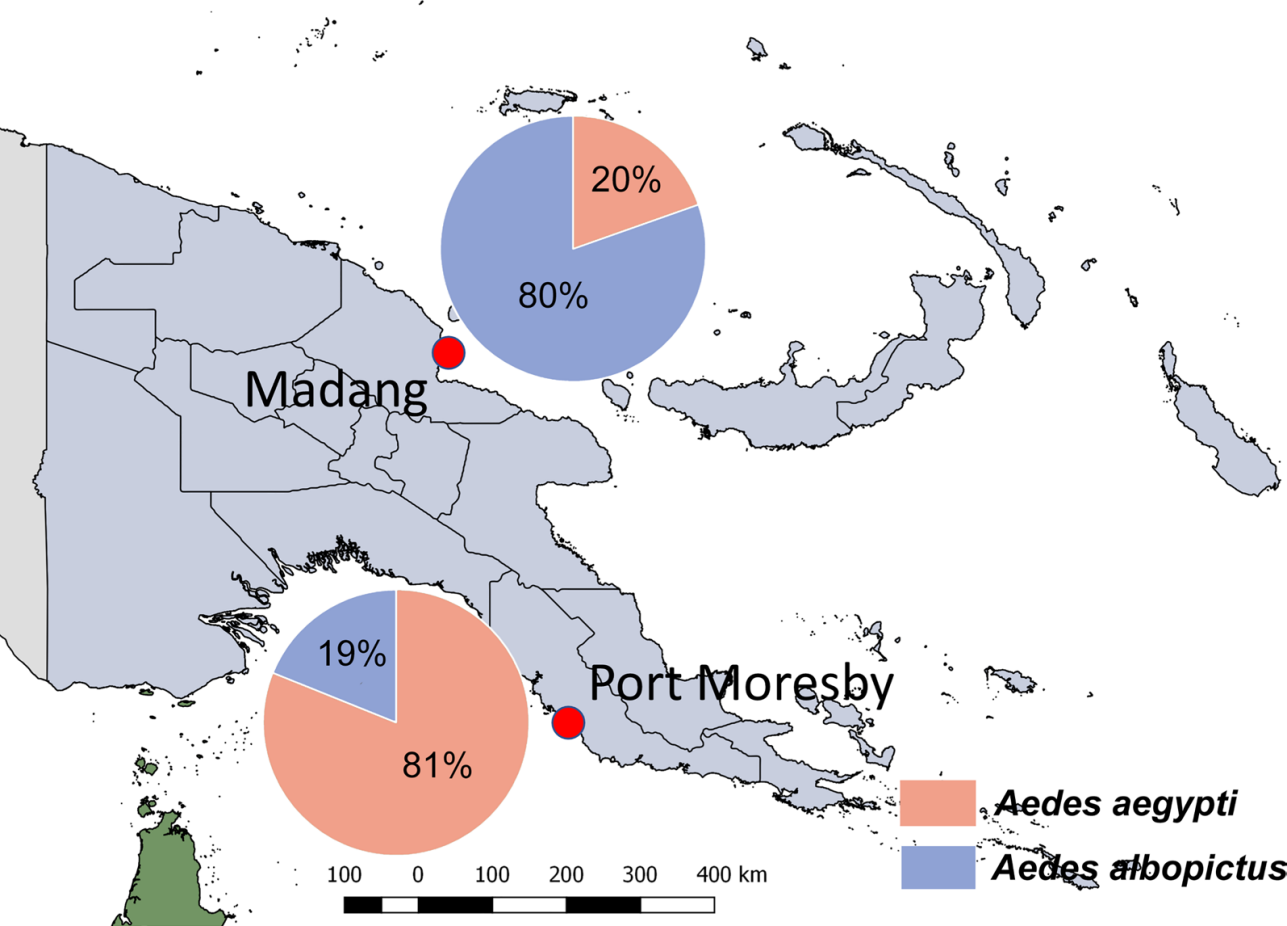
Location of the study sites and relative distribution of *Aedes* spp. in December 2018

In Madang *Ae*. *albopictus* was observed to be the dominant species, whereas in Port Moresby *Ae*. *aegypti* was more abundant. Relative abundance of *Ae. aegypti vs Ae. albopictus* in December 2018, when mosquitoes were trapped in both locations simultaneously, was 19.6 *vs* 80.4% in Madang and 81.1 *vs* 18.9 % in Port Moresby, respectively, as shown in Fig. 1.

### Bioassays

Overall, 1021 female *Ae. aegypti* and *Ae. albopictus* mosquitoes were exposed to insecticides in the bioassays (*n* = 253 *Ae. aegypti* and *n* = 768 *Ae. albopictus*, excluding negative controls done alongside each assay). A total of 26 bioassays were conducted: with deltamethrin (0.05%) (*n* = 10); with DDT (4%) (*n* = 5); with lambda-cyhalothrin (0.05%) (*n* = 5); with bendiocarb (0.1%) (*n* = 3); and with malathion (5%) (*n* = 3). In Port Moresby, only two bioassays with deltamethrin were conducted. The results of the bioassays are presented in Tables 1 and 2 for *Ae. aegypti* and *Ae albopictus*, respectively.

**Table 1:** Bioassay results for *Aedes aegypti*, including samples collected in Madang and Port Moresby. Values are given as proportions and 95% CI [35]

Insecticide, location	Mean knockdown after 60 min (95% CI)	Mean mortality after 24 h (95% CI)
0.05% deltamethrin, Madang (*n* = 106)	0.29 (0.21–0.39)	0.33 (0.25–0.42)
0.05% deltamethrin, Port Moresby (*n* = 46)	0.13 (0.06–0.26)	0.07 (0.02–0.18)
0.05% lambda-cyhalothrin, Madang (*n* = 37)	0.11 (0.04–0.25)	0.32 (0.20–0.49)
0.1% bendiocarb, Madang (*n* = 18)^a^	0.89 (0.66–0.98)	0.89 (0.66–0.98)
4% DDT, Madang (*n* = 13)^a^	0 (0–0.27)	0 (0–0.27)
5% malathion, Madang (*n* = 33)	1.00 (0.88–1.00)	1.00 (0.88–1.00)

^a^The numbers of *Ae. aegypti* mosquitoes tested against Bendiocarb (*n* = 18) and DDT (*n* = 13) in Madang are too low to be statistically robust and these results are included for reason of completeness

*Abbreviations*: n, number of mosquitoes; CI, confidence interval

**Table 2:** Bioassay results for *Aedes albopictus*, including samples collected in Madang and Port Moresby. Values are given as proportions and 95% CI [35]

Insecticide, location	Mean knockdown after 60 min (95% CI)	Mean mortality after 24 h (95% CI)
0.05% deltamethrin, Madang (*n* = 273)	0.98 (0.96–0.99)	1.00 (0.98–1.00)
0.05% deltamethrin, Port Moresby (*n* = 11)^a^	0.92 (0.60–1.00)	0.92 (0.60–1.00)
0.05% lambda-cyhalothrin, Madang (*n* = 135)	1.00 (0.97–1.00)	1.00 (0.97–1.00)
0.1% bendiocarb, Madang (*n* = 123)	1.00 (0.96–1.00)	1.00 (0.96–1.00)
4% DDT, Madang (*n* = 145)^b^	0.62 (0.54–0.70)	0.79 (0.72–0.85)
5% malathion, Madang (*n* = 81)	0.99 (0.93–1.00)	0.96 (0.89–0.99)

^a^The number of mosquitoes tested against deltamethrin in Port Moresby (*n* = 11) is too low to be statistically robust and these results are included for reason of completeness

^b^DDT is known to act more slowly [19] in anophelines, even in susceptible populations. Therefore, we also determined 48 h mortality for most (*n* = 129) *Ae. albopictus* mosquitoes exposed to DDT. Mortality after 48 h was unchanged at 0.77 (0.69–0.83)

*Abbreviations*: n, number of mosquitoes; CI, confidence interval

These data clearly show high levels pyrethroid resistance in both Madang and Port Moresby *Ae*. *aegypti* populations. The *Ae*. *aegypti* population in Port Moresby seems to exhibit a higher level of resistance than that in Madang, with less than 10% of mosquitoes dying during the 24 h holding period after exposure to deltamethrin. Similarly, susceptibility to lambda-cyhalothrin and DDT was greatly reduced (although the number of *Ae. aegypti* exposed to DDT was low). Susceptibility against carbamates (bendiocarb) and organophosphates (malathion) was present in the Madang population (Port Moresby population not tested), but since the number of exposed mosquitoes was low, this result should be treated cautiously.

Susceptibility to DDT of *Ae. albopictus* also appeared to be reduced. The cut-off for confirmed resistance is < 90% mortality over the course of the 24 h holding period [21]. We observed a 24-h mortality of 0.79 (95% CI: 0.72–0.85). *Aedes albopictus* were found to be susceptible to pyrethroids, carbamates and organophosphates.

### Genetic analyses

A total of 55 randomly selected *Ae. aegypti* mosquitoes from Madang were tested for sodium channel mutations using TaqMan® probe assays designed for these sites. The *para* sodium channel or voltage-sensitive sodium channel (*Vssc*) is the target site for both pyrethroid insecticides and DDT [22]. In addition, 48 *Ae. albopictus* mosquitoes were screened by sequencing for *Vssc* mutations at codon 1534, the main site of knockdown resistance mutations in this species [23].

For *Ae*. *aegypti*, three sodium channel mutations, V1016G, F1534C and S989P were found resulting in four composite genotypes. Composite genotype frequencies are summarised in Table 3.

**Table 3:** Frequencies of *Vssc* genotypes of 55 *Aedes aegypti* sampled from Madang, Papua New Guinea

Composite genotypes (V1016G/F1534C/S989P)			
GG/TT/CC	GG/TT/TC	TG/TG/TC	TG/TG/TT
0.64 (35/55)	0.16 (9/55)	0.11 (6/55)	0.09 (5/55)

*Notes*: Wildtype for V1016G is TT whereas homozygous mutant is GG. Wildtype for F1534C is TT whereas homozygous mutant is GG. Wildtype for V1016G is TT whereas homozygous mutant is CC. No (susceptible) wildtype was observed

The most common genotype (frequency = 0.65) consisted of homozygous mutants at codons 1016 and 989 and the wildtype homozygote at codon 1534. This is a common genotype also found in Bali and other locations throughout southeast Asia and the Pacific [24]. It confers resistance to both Type I and Type II pyrethroids.

The second most frequent genotype was the triple heterozygote at V1016G, F1534C and S989P (frequency = 0.16). This is also found commonly in *Ae. aegypti* in other countries in the region and confers a low level of resistance to Type I and II pyrethroids [25].

The remaining two genotypes found were (i) homozygous mutant at codon 1016, wildtype at codon 1534 and heterozygous mutant at codon 989 (frequency: 0.11); and (ii) heterozygous mutants at codons 1016 and 1534, and homozygous wildtype at codon 989 (frequency: 0.09). These genotypes are unusual, but have previously been noted in samples of *Ae. aegypti* from Yogyakarta, Indonesia [24]. Both these genotypes are expected to confer some level of pyrethroid resistance [26]. There were no susceptible wildtype individuals found in the sample and there is no indication that a wildtype haplotype exists in the population.

DNA sequences around codon 1534 were obtained for 36 *Ae*. *albopictus* specimens. None of the specimens showed a mutation within codon 1534. There were some synonymous mutations in other codons which comprised heterozygotes (IUPAC code Y for C/T) in nine individuals. Two individuals were homozygous synonymous mutants at codon 1528 and contained a single base mutation from C to T. The same mutation at this site was observed by Kasai et al. [20], but is not expected to affect susceptibility to pyrethroids. The sequences for the 36 *Ae. albopictus* are provided in Additional file 1.

## Discussion

Insecticide resistance is a threat to vector borne disease control, and pyrethroids are still a widely used insecticide class, e.g. in LLIN for malaria control [27]. Pyrethroid resistance in the highly competent arbovirus vector *Ae*. *aegypti* is widely spread [28], and has also been confirmed in *Ae. albopictus* [28]. In the Asia-Pacific region, reports have confirmed pyrethroid resistance in *Aedes* populations in many countries including Indonesia, but not in Australia [8]. This implies that there is a geographical divide somewhere in the region, between areas where *Aedes* populations are still susceptible and those where *Aedes* populations are resistant [8, 9]. The present data from Port Moresby and Madang indicate that resistance in urban PNG populations may be widely spread, given that we found strong pyrethroid resistance in urban *Ae. aegypti* populations from the north and south coast of the country, and not a single wild type *Ae. aegypti* among the 55 samples tested. Australia has engaged in programs to prevent an incursion of *Ae*. *albopictus via* the Torres Strait for some time [9]. These efforts now seem even more important and should include *Ae. aegypti*, as imported *Ae. aegypti* originating in PNG may well carry resistant alleles. Other incursion routes from Asia also carry this risk [29].

In contrast to the *Ae. aegypti* data, we found no genetic mutations known to convey pyrethroid resistance in the *Ae. albopictus* mosquitoes tested. Moreover, *Ae. albopictus* mosquitoes were susceptible to deltamethrin and lambda-cyhalothrin in bioassays. We did observe reduced susceptibility of *Ae. albopictus* against DDT in Madang using bioassays. Similar results have been found in *Ae*. *albopictus* from India where DDT resistance is detected in bioassays, but is not associated with *Vssc* mutations [30]. DDT resistance in *Ae*. *albopictus* from PNG either may be persisting following previous use of the compound in the country during the malaria eradication era or may be due to incursion of resistant mosquitoes, an occurrence known from other parts of the world [31, 32].

*Aedes aegypti* are more prominent in urban than in rural areas, as these mosquitoes are well adapted to habitats found in urban settings [3]. This may account for the much higher proportion of *Ae*. *aegypti* found in the highly urbanised environment of the national capital Port Moresby compared with the proportion found in Madang (medium sized town, semi-urban). *Aedes albopictus* is known to use a wider range of habitats than *Ae*. *aegypti* and is better adapted to the natural environment than it is to urban areas.

The observed level of pyrethroid resistance in *Ae. aegypti* in the two PNG populations is surprising and unexpected, especially as LLIN usage in cities is lower than in rural areas. For example, no LLIN are distributed in Port Moresby (Tim Freeman, Rotary Against Malaria PNG, primary LLIN distribution agency in PNG, personal communication). No detailed analyses that quantify insecticide usage in PNG in the private sector exist, but to the best of our knowledge, insecticide usage is low. Indirect exposure to pesticides through the agricultural sector seems unlikely, particularly as subsistence farming usually does not involve the use of insecticides. Other sources of insecticide use in PNG are mining companies and other large commercial enterprises focused on resource extraction, as well as hotels and smaller businesses. Little is known about private household-based usage of insecticides, but this could provide further selection pressures. It is therefore possible that the observed pyrethroid resistance is focused on larger population centres or other commercial areas.

Another hypothesis arising from this work is that pyrethroid-resistant *Ae. aegypti* has spread to PNG from neighbouring regions. Human-mediated movement of pyrethroid-resistant *Ae*. *aegypti* is known to occur on a regular basis and mosquitoes with resistance alleles and identified, exotic origins are often intercepted at Australian and New Zealand air and sea ports [33]. It is possible that mosquitoes are transported on marine vessels used, e.g. for fishing and logging, as well as container transport [1] and so incursions to PNG could be possible. Pyrethroid resistance in *Ae*. *aegypti* is thought to be spreading in some parts of Indonesia [34], so potential sources of resistant mosquitoes in the region around PNG are likely to be on the increase. Further surveillance and sampling in PNG is required to map the level of resistance and population genetics of *Ae*. *aegypti* in different parts of the country and identify any potential pathways of incursion.

Limitations of this study include the absence of a susceptible *Aedes* laboratory strain to be used as a control, one of the challenges of working in a remote area with resource limitations. However, our observations were made on samples collected from multiple sites, tested in multiple assays. Bioassay results are also well supported by the genetic analyses presented. The concentrations on the test papers we used were those recommended for anopheline mosquitoes by WHO, whereas the concentrations (unofficially) recommended for *Aedes* mosquitoes are actually lower than those for anophelines [21]. As such our study is unlikely to overestimate the level of resistance but may represent an underestimation.

## Conclusions

The present study identified pyrethroid resistance in *Ae*. *aegypti* in two locations in Papua New Guinea. To our knowledge, this is the first *Aedes* bioassay and *kdr* genotyping data from mosquito populations in Papua New Guinea. Due to presumably low insecticide usage in PNG, it is currently unclear how pyrethroid resistance in PNG arose and is maintained. Studies that investigate and quantify insecticides in the private sector in PNG are urgently needed. Population genetic studies that investigate the relationship between *Aedes* vectors in PNG and neighbouring countries would be useful to determine pathways of spread and incursion routes of resistant populations.

## Additional file

**Additional file 1.** DNA sequences for 36 individuals of *Aedes albopictus* for a small section of the voltage-sensitive sodium channel gene (Vssc) from S6, domain III.

## Data Availability

The datasets used and/or analysed during the current study are available from the corresponding author on reasonable request. Genetic sequences generated during this study are included in Additional file 1.

## Abbreviations

AE: *Aedes*
DDT: dichlorodiphenyltrichloroethane
IRS: indoor residual spraying
KDR: knockdown resistance
LLIN: long-lasting insecticidal nets
PNG: Papua New Guinea
VSSC: voltage-sensitive sodium channel
WHO: World Health Organisation
