# Supplementary material for: Insecticide resistance status of Aedes aegypti and Aedes albopictus mosquitoes in Papua New Guinea
Source: Parasit Vectors. 2019 Jul 3;12:333. doi: 10.1186/s13071-019-3585-6 (PMC6609403; doi:10.1186/s13071-019-3585-6)
Supplement: Supplementary file 1 — Additional file 1. DNA sequences for 36 individuals of Aedes albopictus for a small section of the voltage-sensitive sodium channel gene (Vssc) from S6, domain III. [file 13071_2019_3585_MOESM1_ESM.docx]

***Aedes albopictus* sequences**

DNA sequences for 36 individuals of *Ae.* *albopictus* for a small section of the voltage-sensitive sodium channel gene (*Vssc*)from S6, domain III. Codon 1534 is highlighted in yellow. Sequence is all within the exon in which codon 1534 is located. All individuals show the wildtype state at this codon. Bases unable to be called accurately (N) are highlighted in pink. Synonymous mutations, which comprised heterozygotes (IUPAC code Y for C/T) are highlighted in grey. Two homozygous synonymous mutants were found (both were a mutation from C to T and are highlighted in green).

1 10 20 30 40 50 60

KC152046.1 CTACATGTACCTCTACTTCGTGTTCTTCATCATCTTCGGGTCGTTCTTCACCCTCAACCT

PNG002 -----------------------------TCATCTTCGGGTCGTTCTTCACCCTCAACCT

PNG003 -----------------------------TCATCTTCGGGTCGTTCTTCACCCTCAACCN

PNG004 -----------------------------TCATCTTCGGGTCGTTCTTCACCCTCAACCN

PNG005 -----------------------------TCATCTTCGGGTCGTTCTTCACCCTCAACCT

PNG006 -----------------------------TCATCTTCGGGTCGTTCTTCACCCTCAACCT

PNG007 ------------------------------CATCTTCGGGTCGTTCTTCNCCCTCAACCT

PNG008 -----------------------------TCATCTTCGGGTCGTTCTTCACCCTCAACCT

PNG009 CTACATGTACCTCTACTTYGTGTTCTTCATCATCTTCGGGTCGTTCTTCACCCTCAACCT

PNG010 ---------CCTCTNCTTCGTGTTCTTCATCATCTTCGGGTCGTTCTTCACCCTCAACCT

PNG011 CTACATGTACCTCTACTTCGTGTTCTTCATCATCTTCGGGTCGTTCTTCACCCTCAACCT

PNG012 CTACATGTACCTCTACTTTGTGTTCTTCATCATCTTCGGGTCGTTCTTCACCCTCAACCT

PNG027 ---------------------GTTCTTCATCATCTTCGGGTCGTTCTTCACCCTCAACCT

PNG028 ------------------------CTTCATCATCTTCGGGTCGTTCTTCACCCTCAACCT

PNG029 --------------------------------TCTTCGGGTCGTTCTTCACCCTCAACCT

PNG030 ---------CCTCTACTTCGTGTTCTTCATCATCTTCGGGTCGTTCTTCACCCTYAAYCT

PNG031 CTACATGTACCTCTACTTCGTGTTCTTCATCATCTTCGGGTCGTTCTTCACCC-------

PNG032 CTACATGTACCTCTACTTYGTGTTCTTCATCATCTTCGGGTCGTTCTTCACCC-------

PNG033 CTACATGTACCTCTACTTCGTGTTCTTCATCATCTTCGGGTCGTTCTTCACCCTC-----

PNG034 CTACATGTACCTCTACTTTGTGTTCTTCATCATCTTCGGGTCGTTCTTCAC---------

PNG035 ---------------------GTTCTTCATCATCTTCGGGTCGTTCTTCACCCTCAACCT

PNG037 -----------------------------TCATCTTCGGGTCGTTCTTCACCCTCAACCT

PNG038 --------------------------------TCTTCGGGTCGTTCTTCACCCTYAAYCT

PNG104 CTACATGTACCTCTACTTCGTGTTCTTCATCATCTTCGGGTCGTTCTTCACCCTCAACCT

PNG105 CTACATGTACCTCTACTTYGTGTTCTTCATCATCTTCGGGTCGTTCTTCACCCTCAACCT

PNG106 CTACATGTACCTCTACTTYGTGTTCTTCATCATCTTCGGGTCGTTCTTCACCC-------

PNG107 ---------------------GTTCTTCATCATCTTCGGGTCGTTCTTCACCCTCAACCT

PNG108 -------------------GTGTTCTTCATCATCTTCGGGTCGTTCTTCACCCTCAACCT

PNG109 --------------------------------TCTTCGGGTCGTTCTTCACCCTCAACCT

PNG110 --------------------------------TCTTCGGGTCGTTCTTCACCCTCAACCT

PNG112 --------------------------------TCTTCGGGTCGTTCTTCACCCTCAACCT

PNG113 ------------------------------CATCTTCGGGTCGTTCTTCACCCTCAACCT

PNG114 --------------------------------TCTTCGGGTCGTTCTTCACCCTCAACCT

PNG357 -----------------------------TCATCTTCGGGTCGTTCTTYACCCTCAACCT

PNG358 -----------------------------TCATCTTCGGGTCGTTCTTCACCCTCAACCT

PNG359 -----------------------------TCATCTTCGGGTCGTTCTTCACCCTCAACCT

PNG360 -----------------------------TCATCTTCGGGTCGTTCTTCACCCTCAACCT

60 70 80 90 100 110 120

KC152046.1 GTTCATCGGTGTCATCATCGACAACTTCAACGAGCAGAAGAAGAAAGCCGGTGGCTCGCT

PNG002 GTTCATCGGTGTCATCATCGACAACTTCAACGAGCAGAAGAAGAAAGCCGGTGGCTCGCT

PNG003 GTTCATCGGTGTCATCATCGACAACTTCAACGAGCAGAAGAAGAAAGCCGGTGGCTCGCT

PNG004 GTTCATCGGTGTCATCATCGACAACTTCAACGAGCAGAAGAAGAAAGCCGGTGGCTCGCT

PNG005 NTTCATCGGTGTCATCATCGACAACTTCAACGAGCAGAAGAAGAAAGCCGGTGGCTCGCT

PNG006 GTTCATCGGTGTCATCATCGACAACTTCAACGAGCAGAAGAAGAAAGCCGGTGGCTCGCT

PNG007 GTTCATCGGTGTCATCATCGACAACTTCAACGAGCAGAAGAAGAAAGCCGGTGGCTCGCT

PNG008 GTTCATCGGTGTCATCATCGACAACTTCAACGAGCAGAAGAAGAAAGCCGGTGGCTCGCT

PNG009 GTTCATCGGTGTCATCATCGACAACTTCAACGAGCAGAAGAAGAAAGCCGGTGGCTCGCT

PNG010 GTTCATYGGTGTCATCATCGACAACTTCAACGAGCAGAAGAAGAAAGCCGGTGGCTCNCT

PNG011 GTTCATCGGTGTCATCATCGACAACTTCAACGAGCAGAAGAAGAAAGCCGGTGGCTCGCT

PNG012 GTTCATCGGTGTCATCATCGACAACTTCAACGAGCAGAAGAAGAAAGCCGGTGGCTCGCT

PNG027 GTTCATCGGTGTCATCATCGACAACTTCAACGAGCAGAAGAAGAAAGCCGGTGGCTCGCT

PNG028 GTTCATCGGTGTCATCATCGACAACTTCAACGAGCAGAAGAAGAAAGCCGGTGGCTCGCT

PNG029 GTTCATCGGTGTCATCATCGACAACTTCAACGAGCAGAAGAAGAAAGCCGGTGGCTCGCT

PNG030 GTTCATCGGTGTCATCATCGACAACTTYAACGAGCAGAAGAAGAAAGCCGGTGGCTCGCT

PNG031 ------------------------------------------------------------

PNG032 ------------------------------------------------------------

PNG033 ------------------------------------------------------------

PNG034 ------------------------------------------------------------

PNG035 GTTCATCGGTGTCATCATCGACAACTTCAACGAGCAGAAGAAGAAAGCCGGTGGCTCGCT

PNG037 GTTCATCGGTGTCATCATCGACAACTTCAACGAGCAGAAGAAGAAAGCCGGTGGCTCGCT

PNG038 GTTCATCGGTGTCATCATCGACAACTTYAACGAGCAGAAGAAGAAAGCCGGTGGCTCGCT

PNG104 GTTCATCGGTGTCATCATCGACAACTTCAACGAGCAGAAGAAGAAAGCCGGTGGCTCGCT

PNG105 GTTCATCGGTGTCATCATCGACAACTTCAACGAGCAGAAGAAGAAAGCCGGTGGCTCGCT

PNG106 ------------------------------------------------------------

PNG107 GTTCATCGGTGTCATCATCGACAACTTCAACGAGCAGAAGAAGAAAGCCGGTGGCTCGCT

PNG108 GTTCATCGGTGTCATCATCGACAACTTCAACGAGCAGAAGAAGAAAGCCGGTGGCTCGCT

PNG109 GTTCATCGGTGTCATCATCGACAACTTCAACGAGCAGAAGAAGAAAGCCGGTGGCTCGCT

PNG110 GTTCATCGGTGTCATCATCGACAACTTCAACGAGCAGAAGAAGAAAGCCGGTGGCTCGCT

PNG112 GTTCATCGGTGTCATCATCGACAACTTCAACGAGCAGAAGAAGAAAGCCGGTGGCTCGCT

PNG113 GTTCATCGGTGTCATCATCGACAACTTCAACGAGCAGAAGAAGAAAGCCGGTGGCTCGCT

PNG114 GTTCATCGGTGTCATCATCGACAACTTCAACGAGCAGAAGAAGAAAGCCGGTGGCTCGCT

PNG357 GTTCATCGGTGTCATCATCGACAACTTCAACGAGCAGAAGAAGAAAGCCGGTGGCTCGCT

PNG358 GTTCATCGGTGTCATCATCGACAACTTCAACGAGCAGAAGAAGAAAGCCGGTGGCTCGCT

PNG359 GTTCATCGGTGTCATCATCGACAACTTCAACGAGCAGAAGAAGAAAGCCGGTGGCTCGCT

PNG360 GTTCATCGGTGTCATCATCGACAACTTCAACGAGCAGAAGAAGAAAGCCGGTGGCTCGCT

120 130 140 150 160 170 180

KC152046.1 GGAAATGTTCATGACGGAGGATCAGAAAAAGTACTACAACGCAATGAAAAAGATGGGCTC

PNG002 GGAAATGTTCATGACGGAGGATCAGAAAAAGTACTACAACGCAATGAAAAAGATGGGCTC

PNG003 GGAAATGTTCATGACGGAGGATCAGAAAAAGTACTACAACGCAATGAAAAAGATGGGCTC

PNG004 GGAAATGTTCATGACGGAGGATCAGAAAAAGTACTACAACGCAATGAAAAAGATGGGCTC

PNG005 GGAAATGTTCATGACGGAGGATCAGAAAAAGTACTACAACGCAATGAAAAAGATGGGCTC

PNG006 GGAAATGTTCATGACGGAGGATCAGAAAAAGTACTACAACGCAATGAAAAAGATGGGCTC

PNG007 GGAAATGTTCATGACGGAGGATCAGAAAAAGTACTACAACGCAATGAAAAAGATGGGCTC

PNG008 GGAAATGTTCATGACGGAGGATCAGAAAAAGTACTACAACGCAATGAAAAAGATGGGCTC

PNG009 GGAAATGTTCATGACGGAGGATCAGAAAAAG-----------------------------

PNG010 GGAAATGTTCATGACGGAGGATCAGAAAAAGTACTACAACGCAATGAAAAAGATGGGCTC

PNG011 GGAAATGTTCATGACGGAGGATCAGAAAAAGTACTACAACGCAATGAAAAAGATGGGCTC

PNG012 GGAAATGTTCATGACGGAGGATCAGAAAAAGTACTACAACGCAATGAAAAAGATGGGCTC

PNG027 GGAAATGTTCATGACGGAGGATCAGAAAAAGTACTACAACGCAATGAAAAAGATGGGCTC

PNG028 GGAAATGTTCATGACGGAGGATCAGAAAAAGTACTACAACGCAATGAAAAAGATGGGCTC

PNG029 GGAAATGTTCATGACGGAGGATCAGAAAAAGTACTACAACGCAATGAAAAAGATGGGCTC

PNG030 GGAAATGTTCATGACGGAGGATCAGAAAAAGTACTACAACGCAATGAAAAAGATGGGCTC

PNG031 ------------------------------------------------------------

PNG032 ------------------------------------------------------------

PNG033 ------------------------------------------------------------

PNG034 ------------------------------------------------------------

PNG035 GGAAATGTTCATGACGGAGGATCAGAAAAAGTACTACAACGCAATGAAAAAGATGGGCTC

PNG037 GGAAATGTTCATGACGGAGGATCAGAAAAAGTACTACAACGCAATGAAAAAGATGGGCTC

PNG038 GGAAATGTTCATGACGGAGGATCAGAAAAAGTACTACAACGCAATGAAAAAGATGGGCTC

PNG103 -----------TGACGGAGGATCAGAAAAAGTACTACAACGCAATGAAAAAGATGGGCTC

PNG104 GGAAATGTTCATGACGGAGGATCAGAAAAAGTACTACAACGCAATGAAAAAGATGGGCTC

PNG105 GGAAATGTTCATGACGGAGGATCAGAAAAAGTACTACAACGCAATGAAAAAGATGGGCTC

PNG106 ------------------------------------------------------------

PNG107 GGAAATGTTCATGACGGAGGATCAGAAAAAGTACTACAACGCAATGAAAAAGATGGGCTC

PNG108 GGAAATGTTCATGACGGAGGATCAGAAAAAGTACTACAACGCAATGAAAAAGATGGGCTC

PNG109 GGAAATGTTCATGACGGAGGATCAGAAAAAGTACTACAACGCAATGAAAAAGATGGGCTC

PNG110 GGAAATGTTCATGACGGAGGATCAGAAAAAGTACTACAACGCAATGAAAAAGATGGGCTC

PNG112 GGAAATGTTCATGACGGAGGATCAGAAAAAGTACTACAACGCAATGAAAAAGATGGGCTC

PNG113 GGAAATGTTCATGACGGAGGATCAGAAAAAGTACTACAACGCAATGAAAAAGATGGGCTC

PNG114 GGAAATGTTCATGACGGAGGATCAGAAAAAGTACTACAACGCAATGAAAAAGATGGGCTC

PNG357 GGAAATGTTCATGACGGAGGATCAGAAAAAGTACTACAACGCAATGAAAAAGATGGGCTC

PNG358 GGAAATGTTCATGACGGAGGATCAGAAAAAGTACTACAACGCAATGAAAAAGATGGGCTC

PNG359 GGAAATGTTCATGACGGAGGATCAGAAAAAGTACTACAACGCAATGAAAAAGATGGGCTC

PNG360 GGAAATGTTCATGACGGAGGATCAGAAAAAGTACTACAACGCAATGAAAAAGATGGGCTC

**GenBank Accession Numbers for the above *Ae. albopictus* sequences**

All sequences were uploaded to GenBank. The accession numbers are provided below.

BankIt2227903 PNG002 MK991980

BankIt2227903 PNG003 MK991981

BankIt2227903 PNG004 MK991982

BankIt2227903 PNG005 MK991983

BankIt2227903 PNG006 MK991984

BankIt2227903 PNG007 MK991985

BankIt2227903 PNG008 MK991986

BankIt2227903 PNG009 MK991987

BankIt2227903 PNG010 MK991988

BankIt2227903 PNG011 MK991989

BankIt2227903 PNG012 MK991990

BankIt2227903 PNG027 MK991991

BankIt2227903 PNG028 K991992

BankIt2227903 PNG029 MK991993

BankIt2227903 PNG030 MK991994

BankIt2227903 PNG031 MK991995

BankIt2227903 PNG032 MK991996

BankIt2227903 PNG033 MK991997

BankIt2227903 PNG034 MK991998

BankIt2227903 PNG035 MK991999

BankIt2227903 PNG037 MK992000

BankIt2227903 PNG038 MK992001

BankIt2227903 PNG104 MK992002

BankIt2227903 PNG105 MK992003

BankIt2227903 PNG106 MK992004

BankIt2227903 PNG107 MK992005

BankIt2227903 PNG108 MK992006

BankIt2227903 PNG109 MK992007

BankIt2227903 PNG110 MK992008

BankIt2227903 PNG112 MK992009

BankIt2227903 PNG113 MK992010

BankIt2227903 PNG114 MK992011

BankIt2227903 PNG357 MK992012

BankIt2227903 PNG358 MK992013

BankIt2227903 PNG359 MK992014
